# A new *Pseudophoxinus* (Teleostei, Cyprinidae) species from Asi River Drainage (Turkey)

**DOI:** 10.3897/zookeys.411.6833

**Published:** 2014-05-23

**Authors:** Fahrettin Küçük, Salim Serkan Güçlü

**Affiliations:** 1 Süleyman Demirel University, Eastern Campus, Eğirdir Fisheries Faculty, Isparta-Turkey

**Keywords:** Anatolia, Asi River, freshwater fishes, Leuciscinae, taxonomy

## Abstract

*Pseudophoxinus turani* **sp. n.** is described from the İncesu Spring (Hassa-Hatay) drainage of Asi River, Turkey. It is distinguished from other Eastern Mediterranean Region *Pseudophoxinus* species by a combination of characters: lateral line incomplete, with 12–25 (commonly 16–21) perforated scales and 38–46+2-3 scales in lateral series (commonly 41–44+2-3); 10–11 scale rows between the lateral line and dorsal-fin origin; 3–4 scale rows between the lateral line and the pelvic–fin origin; dorsal fin with 7½ branched rays; anal fin commonly with 7½ branched rays; 8-11gill rakers on the first branchial arch; dorsal profile markedly convex with marked hump at the nape, ventral profile less convex than dorsal profile; a small, irregular, black blotch on the base of the caudal fin; mouth terminal, with slightly distinct chin, its corner not reaching vertical through anterior margin of eye; snout somewhat long, with rounded tip; and its length greater than eye diameter.

## Introduction

Members of the cyprinid genus *Pseudophoxinus* are small minnows mostly found in cold springs, slow-flowing waters and clean lakes (Küçük et al. 2013). Speciation and phylogenetic relationships within the genus in Anatolia were first studied by Hrbek et al. (2004), who argued that the Tohma Stream population (Fırat River drainage) (originally published as *Pseudophoxinus* new species, now *Pseudophoxinus firati*) and *Pseudophoxinus kervillei* from the Asi River form a separate lineage distinct from all other congeners in Central Anatolia, the Lakes Region and Büyük Menderes basins. A more comprehensive and detailed study based on mitochondrial and nuclear DNA data corroborates the hypothesis that the genus *Pseudophoxinus* is represented in Anatolia by two monophyletic lineages (Central Anatolian and Eastern Mediterranean Region clades) and noted uncertainty in Anatolian *Pseudophoxinus* species boundaries (Perea et al. 2010).

The original description of the Eastern Mediterranean Region species *Pseudophoxinus kervillei* by Pellegrin (1911: 109–110, 1928: 120–121) includes the following information: lateral line incomplete, 37–42 scales in lateral series, 9–10 scale rows between lateral line and dorsal-fin origin, 7–8 scale rows between lateral line and the pelvic-fin origin, D 11, A 10, P 13, V 8. The distribution area originally given as Asi River, Adana and Islahiye near Osmaniye (Pellegrin 1928: 121), is restricted to Jordan, Litani and Asi rivers basins according to Krupp (1985), who described a *Phoxinellus* (=*Pseudophoxinus*) sp. from Hupnik Stream (near Islahiye, 22 km southeast Gaziantep), a drainage of Asi River, with a shorter lateral line (0–18 scales vs. 4–27 in *Pseudophoxinus kervillei*), more gill rakers and more branched anal-fin rays. Since then this population hasn’t been studied and couldn’t be relocated at site during our surveys in June 2012 and October 2013. Bogutskaya (1997: 177–178) wrote that the *Pseudophoxinus* specimens from the Ceyhan River (ZMH 1103: Hamburg Zoological Museum, now *Pseudophoxinus zekayi*) and İncesu Spring (ZMH 8001) (mentioned in the original text as Seyhan tributary, although it is a tributary of Asi) differed from Asi River *Pseudophoxinus kervillei* in having more scales in the lateral series (55–60 vs. 35–50 in *Pseudophoxinus kervillei*) and presence of 25–34 perforate scales (4–17 in *Pseudophoxinus kervillei*). Perea et al. (2010) also tentatively identified the *Pseudophoxinus* population from İncesu Spring (Hassa, Hatay) as *Pseudophoxinus* cf. *kervillei* due to molecular distinction of the population from *Pseudophoxinus kervillei* (Asi River).

Our evaluation of morphological features and the distribution areas of Eastern Mediterranean Region species *Pseudophoxinus firati*, *Pseudophoxinus kervillei*, *Pseudophoxinus zeregi* and *Pseudophoxinus zekayi* indicates that the İncesu (Hassa, Hatay) specimens represent a new species distinct from the Asi River basin *Pseudophoxinus kervillei*, which is described below.

## Materials and methods

Fish specimens were collected by pulsed DC electrofishing equipment, killed by over anaesthetization and fixed in 5% formalin. Material is deposited in: IFC-ESUF, Inland Fishes Collection, Eğirdir Fisheries Faculty of Süleyman Demirel University. Counts and measurements follow Kottelat and Freyhof (2007). All measurements were point to point and made with digital calipers (0.01 mm sensitive). Other metrics include head width_1_ (the distance between the anterior eye margins), head width_2_ (the distance between the posterior eye margins), head width_3_ (head width at the nape), head depth_1_ (head depth through the eye), head depth_2_ (head depth at the nape), and the snout width (measured at level of the nostrils). The perforated lateral-line scales were counted from the anteriormost scale (the first one to touch the shoulder girdle) to the posteriormost one; scales in lateral series were counted along the midlateral line from the first one to touch the shoulder girdle to the last scale at the end of the hypural complex; scales on the caudal fin itself are indicated by “+”; the last two branched dorsal and anal fin rays articulating on a single pterygiophore were counted as 1½. The vertebral counts were obtained from radiographs and counted following Naseka (1996); abdominal vertebrae were counted from the first Weberian vertebra to the one just anterior to the first caudal vertebra (the most anterior vertebra that has a fully developed haemal spine; the last complex vertebra bearing hypurals was included in the count of total and caudal vertebrae. Cephalic sensory canals were studied under a stereomicroscope.

The morphometric characters of the two species of *Pseudophoxinus* from Turkey were compared by Principal Component Analysis (PCA) using a covariance matrix on log–transformed measurements and counts with the software package PAST version 1.8 (Hammer et al. 2001).

**Figure 1.**
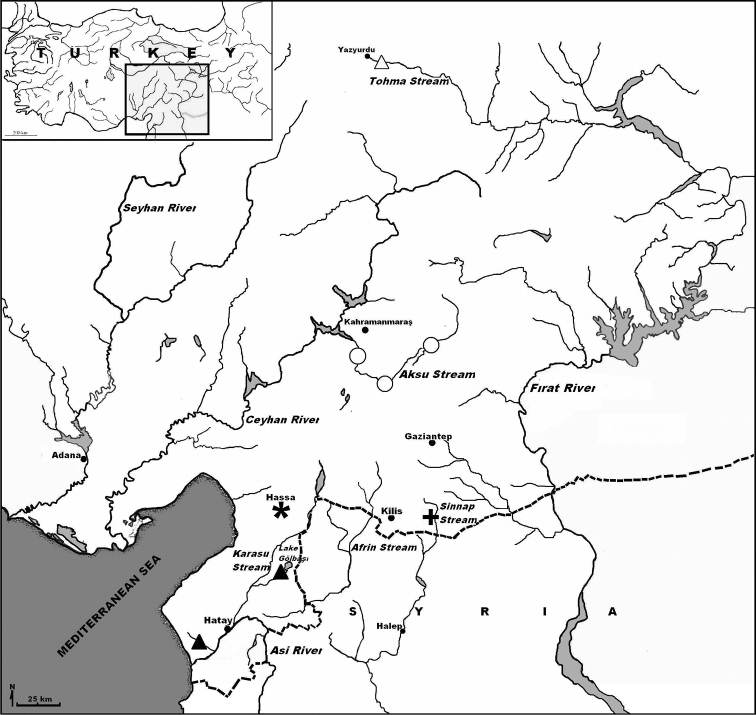
Map showing localities of Eastern Mediterranean Region *Pseudophoxinus* species group (△ *Pseudophoxinus firati*, ▲ *Pseudophoxinus kervillei*, ✱ *Pseudophoxinus turani* sp. n., ○ *Pseudophoxinus zekayi*, ➕ *Pseudophoxinus zeregi*).

## Results

### Pseudophoxinus turani sp. n.

http://zoobank.org/98DF0D2B-6917-44DA-8EAC-E524C0BEB787

http://species-id.net/wiki/Pseudophoxinus_turani

Figures 2 , 3

#### Holotype.

IFC-ESUF 03-1002, 71.3 mm SL; Turkey, Hatay Prov., Hassa Country, İncesu Spring, Asi River drainage, 36°47.36'N, 36°30.48'E, 20 October 2013, coll. F. Küçük and A. Küçük.

#### Paratypes.

IFC-ESUF 03-1003, 20, 52.1-93.4 mm SL, same as holotype.

#### Diagnosis.

*Pseudophoxinus turani* is distinguished from all other species of Eastern Mediterranean Region *Pseudophoxinus* (*Pseudophoxinus firati*, *Pseudophoxinus kervillei*, *Pseudophoxinus zeregi*, *Pseudophoxinus zekayi*) by the following unique combination of characters: head short, its length 26–28% SL, approximately equal to or slightly shorter than body depth; mouth terminal, with slightly distinct chin, its corner not reaching vertical through anterior margin of eye; eye small, its diameter 25–29% HL, smaller than snout length; lateral line incomplete, with 12–25 (commonly 16–21) perforated scales and 38-46+2-3 scales in lateral series (commonly 41–44 +2-3); 10–11 scale rows between lateral line and dorsal-fin origin; 3–4 scale rows between lateral line and the pelvic-fin origin; 8-11(rarely 13) gill rakers on the first branchial arch; pharyngeal teeth 5–4, slightly serrated and hooked at tip.

#### Description

**Figure 2.**
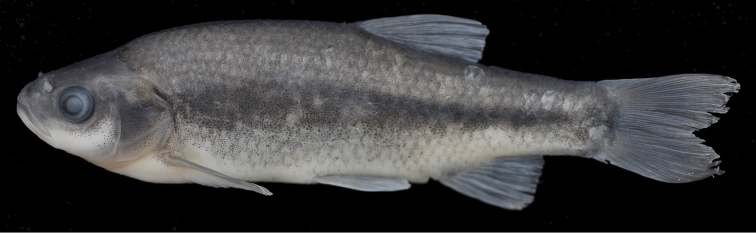
*Pseudophoxinus turani* sp. n. holotype, IFC-ESUF 03-1002, 71.3 mm SL, Turkey: Hatay prov.: Hassa, İncesu Spring, Asi River drainage.

**Figure 3.**
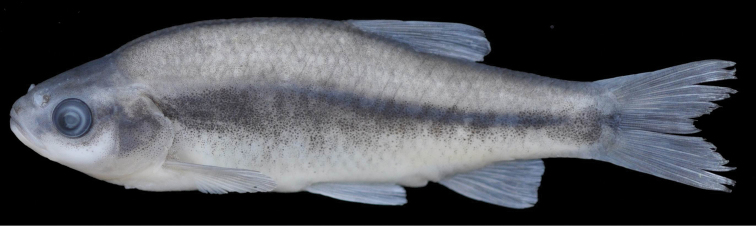
*Pseudophoxinus turani* sp. n. paratype, IFC-ESUF 03-1003, 66.0 mm SL, Turkey: Hatay prov.: Hassa, İncesu Spring, Asi River drainage.

(See Figs 2, 3 for general appearance and Tables 1, 2 for morphometric and meristic data). Body deep, its depth at dorsal-fin origin 26–29% SL, mean 27.8, and laterally compressed. Dorsal profile markedly convex with marked hump at nape, ventral profile less convex than dorsal profile. Dorsal-fin origin situated behind base of pelvic-fin. Predorsal length 56–60% SL, mean 58.2 and prepelvic length 51–54% SL, mean 52.4. Head short, its length 26–28% SL, mean 26.9, approximately equal to or slightly shorter than body depth, upper profile straight or slightly convex on interorbital area and markedly convex on snout. Mouth terminal, with slightly marked chin, its corner not reaching vertical through anterior margin of eye. Eye small, its diameter 25–29% HL, mean 26.6. Snout somewhat long, with rounded tip, its length 27–31% HL, mean 30.4, greater than eye diameter. Caudal-peduncle length 17–20% SL, mean 18.4; caudal-peduncle length 1.3–1.7, mean 1.5, times its depth. Lateral line incomplete, commonly not reaching the level of anus, 12–25 perforated scales, 38–46+2-3 scales in lateral series. Dorsal fin with 3 simple and 7½ branched rays, outer margin straight or slightly convex. Anal fin with 3 simple and 6½ (2 specimens)–7½ (19 specimens) branched rays, outer margin straight or slightly convex. Pectoral fins with 11–12 (rarely 13) branched rays, outer margin convex. Pelvic fins with 6 branched rays, outer margin convex. Caudal fin forked, lobes rounded. No pelvic axillary lobe and keel between posterior pelvic fin base and anus. Pharyngeal teeth 5–4, slightly serrated, hooked at tip. Gill rakers short, with 8–11 (rarely 13) on outer side of first gill arch. Scales oval, with numerous radii posteriorly. Total vertebrae 36–38, 21–22 abdominal and 16-17 caudal vertebrae, vertebral formulae: 36–38:20–21+16–17.

**Table 1.** Morphometry of *Pseudophoxinus turani* sp. n. (holotype IFC-ESUF 03-1002, paratypes IFC-ESUF 03-1003, n=20 )and *Pseudophoxinus kervillei* (IFC-ESUF 03-0987, n=21).

	*Pseudophoxinus turani*		*Pseudophoxinus kervillei*
	Holotype	Paratypes	
**In percent of standard length**			
Head Length	26.8	26.0-27.5 (26.9) ±0.001	24.8-29.9 (27.5) ±0.003
Body depth of dorsal- fin origin	26.0	25.9-29.2 (27.8) ±0.001	24.9-29.5 (27.8) ±0.003
Predorsal distance	57.8	55.9-59.6 (58.2) ±0.002	54.9-59.5 (56.9) ±0.003
Prepelvic distance	52.3	50.7-54.3 (52.4) ±0.002	50.9-54.7 (52.8) ±0.003
Preanal distance	73.5	70.8-76.2 (73.6) ±0.003	70.3-77.9 (72.8) ±0.006
Distance between pectoral and anal-fin origins	47.7	45.4-49.5 (48.1) ±0.003	43.7-49.7 (46.6) ±0.005
Distance between pectoral and pelvic-fin origins	25.5	24.1-26.7 (25.9) ±0.002	21.8-27.2 (23.9) ±0.004
Distance between pelvic and anal-fin origins	21.7	20.5-23.9 (22.1) ±0.002	20.7-23.9 (22.4) ±0.003
Dorsal fin depth	22.4	21.9-24.8 (23.5) ±0.001	19.6-25.3 (22.7) ±0.005
Dorsal fin length	12.4	11.7-13.9 (12.9) ±0.001	11.7-12.9 (12.5) ±0.003
Anal fin depth	16.9	16.8-19.3 (18.1) ±0.001	16.1-18.9 (17.8) ±0.002
Anal fin length	11.2	10.9-12.1 (11.3) ±0.004	10.1-13.6 (11.6) ±0.003
Pectoral fin length	20.1	16.2-20.8 (19.3) ±0.002	17.5-23.1 (19.0) ±0.004
Pelvic fin length	17.1	14.7-19.9 (17.8) ±0.003	16.3-18.2 (16.9) ±0.001
Caudal peduncle length	20.1	17.0-20.1 (18.4) ±0.002	15.2-20.3 (17.5) ±0.004
Caudal peduncle depth	12.5	11.7-13.9 (12.8) ±0.001	10.9-12.9 (12.1) ±0.002
**In percent of head length**			
Snout length	28.5	26.8-31.5 (30.4) ±0.003	25.7-31.4 (28.0) ±0.005
Eye diameter	25.7	24.8-29.3 (26.6) ±0.003	26.9-32.7 (29.5) ±0.005
Interorbitaldistance	36.7	36.2-40.9 (38.9) ±0.003	35.1-42.3 (38.4) ±0.006
Head width 1	36.4	32.6-38.8 (36.3) ±0.004	29.6-35.7 (32.7) ±0.005
Head width 2	47.4	47.4-52.4 (50.5) ±0.002	44.8-52.1 (48.4) ±0.006
Head width 3	53.5	51.6-58.1 (54.9) ±0.003	50.1-56.4 (53.2) ±0.006
Head depth 1	56.5	54.4-63.8 (57.1) ±0.005	53.1-56.3 (55.1) ±0.005
Head depth 2	77.6	74.5-82.8 (78.8) ±0.007	71.8-79.0 (74.2) ±0.006
Internostril distance	20.9	20.2-24.8 (22.2) ±0.003	19.2-25.4 (22.2) ±0.004
Mouth width	26.5	23.1-29.4 (27.1) ±0.003	23.3-27.7 (25.2) ±0.004
Lower jaw length	36.9	34.9-42.7 (38.6) ±0.004	35.0-41.2 (38.2) ±0.004

**Table 2.** Meristic features of the Eastern Mediterranean Region *Pseudophoxinus* species group (from comparative material).

Species	Lateral series scales	Lateral line scales	Pharyngeal teeth	Gill rakers	Vertebral formula
*Pseudophoxinus firati*	41–49+1–2	35–51	5–5 (4)	6–7	37–38: 22+16–17
*Pseudophoxinus kervillei*	37–44+2–3	4–17	5–4	7–8	35–36:19–20+16–17
*Pseudophoxinus turani* sp. n.	**38–46+2–3**	**12–25**	**5–4**	**8–11(13)**	**36–38:21–22+16–17**
*Pseudophoxinus zekayi*	40–46+1–2	36–43	5–5	7–9	37–39:21–22+15–17
*Pseudophoxinus zeregi*	54–59+2–3	47–53	5–4	7–9	36–38:19–21+16–17

#### Sexual dimorphism.

There is no sexual dimorphism between males and females

#### Coloration.

Ground color of formalin-preserved adults and juveniles dark grey on back and upper part of flank, yellowish on lower part of flank and belly. Dark grey stripe (its width 1 to 2 times eye diameter) on the middle of flank from posterior margin of operculum to caudal peduncle, distinct in both anterior and posterior parts of body. Caudal, dorsal and anal fins grey; pectoral and pelvic fins light grey. Black spots present on rays of all fins, additionally on the dorsal-fin base. A small black blotch of pigment present on caudal-fin base. Peritoneum grayish to blackish, with numerous crystal-shaped black spots.

#### Etymology.

The species is named after Davut Turan (Recep Tayyip Erdoğan University, Rize), in appreciation for his contributions to our knowledge of the fish fauna of Anatolia.

## Discussion

As stated by Küçük et al. (2013), unlike the central and western Anatolian species, which differ from one another in complex morphological features, the Eastern Mediterranean *Pseudophoxinus* species (mentioned as Levant species) are morphologically quite similar with the exception of *Pseudophoxinus zekayi*, which differs from the others in having a complete lateral line.

Below we compare *Pseudophoxinus turani* from the İncesu Spring, a drainage of Asi River, with the Eastern Mediterranean region *Pseudophoxinus* species group: *Pseudophoxinus kervillei* from Gölbaşı Lake (Asi River drainage), *Pseudophoxinus zekayi* from Aksu Stream (Ceyhan River drainage), *Pseudophoxinus zeregi* from Sinnap Stream (Kuveik River drainage), and *Pseudophoxinus firati* from Tohma Stream (Fırat River drainage) (Pellegrin 1911; 1928; Bogutskaya 1997; Bogutskaya et al. 2007). *Pseudophoxinus turani* is easily distinguished from *Pseudophoxinus kervillei* by its terminal mouth (vs. slightly superior) and rounded snout (vs. slightly rounded). It is further distinguished from *Pseudophoxinus kervillei* by having more gill rakers on the outer side of the first gill arch (8–11, rarely 13, vs.7–8), usually more lateral-line scales (12–25, vs. 4–17), more abdominal vertebrae (21–22, vs. 19–20), usually more total vertebrae (36–38, vs. 35–36) and sometimes fewer branched pelvic-fin rays (6 vs. 6–7). Besides the differences listed above *Pseudophoxinus turani* has a smaller eye diameter and longer snout than *Pseudophoxinus kervillei*.

*Pseudophoxinus turani* and *Pseudophoxinus kervillei* were also compared by Principal Component Analysis (PCA) using 27 morphometric characters. The PCA clearly separated *Pseudophoxinus turani* from *Pseudophoxinus kervillei* (Fig. 5). Variables loading on the first metric PC I–II are given in Table 3.

**Figure 4.**
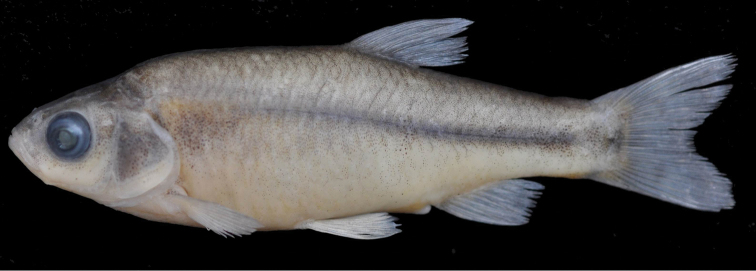
*Pseudophoxinus kervillei* IFC-ESUF 03-0987, 73.2 mm SL, Turkey: Hatay prov.: Lake Gölbaşı, Asi River drainage.

**Figure 5.**
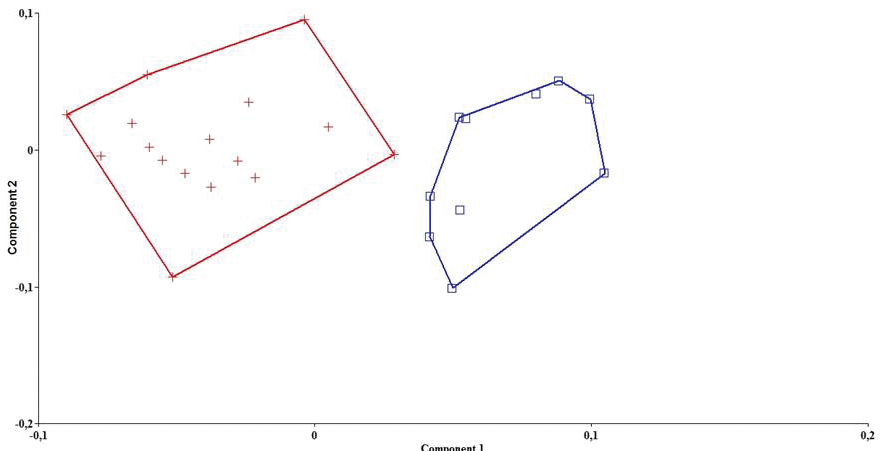
A scatter plot of the scores of the first two principal components (PC I, PC II) for 28 specimens of two *Pseudophoxinus* species, *Pseudophoxinus turani* sp. n. (+) and *Pseudophoxinus kervillei* (□), based on 27 morphometric characters.

**Table 3.** Character loading on principal components I–II for 27 measurements taken on 28 specimens of two *Pseudophoxinus* species (*Pseudophoxinus turani* sp. n. and *Pseudophoxinus kervillei*).

Morphometric features		
In percent of standard length	PC I	PCA II
Head Length	0.052	-0.162
Body depth of dorsal- fin origin	-0.030	-0.144
Predorsal distance	-0.104	-0.043
Prepelvic distance	0.006	-0.048
Preanal distance	-0.055	-0.096
Distance between pectoral and anal-fin origins	-0.128	-0.143
Distance between pectoral and pelvic-fin origins	-0.296	-0.084
Distance between pelvic and anal-fin origins	0.011	-0.259
Dorsal fin depth	-0.161	-0.101
Dorsal fin length	-0.099	0.021
Anal fin depth	-0.060	0.074
Anal fin length	0.274	0.166
Pectoral fin length	-0.106	-0.306
Pelvic fin length	-0.231	0.095
Caudal peduncle length	-0.243	0.132
Caudal peduncle depth	-0.275	-0.208
**In percent of head length**		
Snout length	-0.327	0.138
Eye diameter	0.363	0.261
Interorbitaldistance	-0.022	0.209
Head width 1	-0.426	0.077
Head width 2	-0.130	0.124
Head width 3	-0.128	0.076
Head depth 1	-0.172	0.129
Head depth 2	-0.249	0.181
Internostril distance	0.027	0.356
Mouth width	-0.136	0.491
Lower jaw length	0.006	0.253

*Pseudophoxinus turani* is distinguished from *Pseudophoxinus zeregi* by having fewer lateral-line scales (12–25, vs. 47–53), fewer lateral series scales (38–46+2–3, vs. 54–59+2–3), fewer scales between lateral line and dorsal-fin origin (10–11, vs.11–13) and usually more gill rakers on first gill arch (8–11, rarely 13, vs. 7–9). In *Pseudophoxinus turani*, membranes of fins are grey and rays have black spots, while in *Pseudophoxinus zeregi* membrane of fins are hyaline and rays lack black spots.

*Pseudophoxinus turani* is clearly separable from *Pseudophoxinus zekayi* by having an incomplete lateral line (vs. complete), fewer lateral-line scales (12–25 vs. 36–44), fewer pharyngeal teeth (5–4, vs. 5–5) and a longer snout (28.5–31.7 % HL, mean 30.3, vs. mean 26.01 % HL). In *Pseudophoxinus turani*, eye diameter is smaller than snout length while in *Pseudophoxinus zekayi*, eye diameter is equal to or greater than snout length.

*Pseudophoxinus turani* is distinguished from *Pseudophoxinus firati* by having fewer lateral-line scales (12–25 vs. 35–51) and more gill rakers on the outer side of the first gill arch (8–11, rarely 13, vs. 6–7). *Pseudophoxinus turani* is also distinguished from *Pseudophoxinus firati* by having a black spot on base of caudal fin (vs. lacking), slightly shorter head length (26.0–27.5, mean 26.9 %SL, vs. mean 28.6), 3 simple dorsal-fin rays (vs. commonly 4), and more scales between lateral-line and dorsal-fin origins (10–11, vs. commonly 9).

Our data on meristic features of the Eastern Mediterranean *Pseudophoxinus* (Table 2) are largely compatible with previously published counts: Heckel (1843) counted 57–66 perforated scales on the lateral line of the type species, *Pseudophoxinus zeregi*, from the Kuveik River near Aleppo, whereas we counted 47–53 scales in our material from Sinnap Stream (Kuveik River drainage) (We believe that Heckle (1843) were counted scales in lateral series along the midlateral line). Lateral series and lateral line scale counts of *Pseudophoxinus kervillei* are compatible with that of Pellegrin (1928) and Küçük et al. (2013). Meristic data of *Pseudophoxinus firati* and *Pseudophoxinus zekayi* are also found largely to be in conformity with Bogutskaya et al. (2007) and Küçük et al. (2013).

## Comparative material (all from Turkey)

*Pseudophoxinus firati*: IFC-ESUF 03-0999, 12, 34.6–51.7 mm SL, Sivas prov.: Fırat River drainage, Yazyurdu, M.A. Atalay, 04 August 2004; 03-1001, 4 (paratypes),41.2–47.7 mm SL, Sivas prov: Fırat River drainage, Tohma Stream at Yazyurdu, T. Hrbek, K.N. Stölting, R.H. Wildekamp and A. Meyer, ? April 2000.

*Pseudophoxinus kervillei*: IFC-ESUF 03-0987,26, 60.7–84.9 mm SL, Hatay prov.: Lake Gölbaşı-Kırıkhan, F.Küçük, D.Turan, S.S. Güçlü, 01 July 2012; 03-0988, 25, 27.4–56.0 mm SL, Hatay prov.: Meydan Village-Samandağ, F.Küçük, D.Turan, S.S. Güçlü, H.Temizkan, 30 June 2012.

*Pseudophoxinus zekayi*: IFC-ESUF 03-1007, 32, 28.5–62.1 mm SL, Kahramanmaraş prov.: Aksu Stream, F.Küçük, D.Turan, S.S. Güçlü, 29 June 2012.

*Pseudophoxinus zeregi*: IFC-ESUF 03-1011, 47, 33.9–64.5 mm SL, Kilis prov.: Sinnap Stream, F.Küçük, D.Turan, S.S. Güçlü, M. Kamer, C. Kaya, 04 November 2012 and 26 June 2013; IFC-ESUF 03-1012, 4, 36.3–64.5 mm SL, Kilis prov.: Sinnap Stream, İnanlı Village, C. Kaya, E. Gürlek, 26 June 2013.

## Supplementary Material

XML Treatment for Pseudophoxinus turani
